# Targeting Persistent Biofilm Infections: Reconsidering the Topography of the Infection Site during Model Selection

**DOI:** 10.3390/microorganisms10061164

**Published:** 2022-06-06

**Authors:** Ilana Kolodkin-Gal, Malena Cohen-Cymberknoh, Gideon Zamir, Igor Tsesis, Eyal Rosen

**Affiliations:** 1 Department of Plant Pathology and Microbiology, The Hebrew University of Jerusalem, Rehovot 7610001, Israel; 2 Pediatric Pulmonary Unit and Cystic Fibrosis Center, Hadassah Medical Center and Faculty of Medicine, Hebrew University of Jerusalem, Jerusalem 9112001, Israel; malena@hadassah.org.il; 3 Department of Experimental Surgery, Hadassah Hebrew University Medical School, Jerusalem 9112001, Israel; rgideonz@hadassah.org.il; 4 Department of Endodontics, Goldschleger School of Dental Medicine, Sackler Faculty of Medicine, Tel Aviv University, Tel Aviv 6997801, Israel; 5 Center for Nanoscience and Nanotechnology, Tel Aviv University, Tel Aviv 6997801, Israel

**Keywords:** experimental models, biofilm, infection, dentin, *Enterococcus faecalis*, *Pseudomonas aeruginosa*

## Abstract

The physiology of an organism in the environment reflects its interactions with the diverse physical, chemical, and biological properties of the surface. These principles come into consideration during model selection to study biofilm–host interactions. Biofilms are communities formed by beneficial and pathogenic bacteria, where cells are held together by a structured extracellular matrix. When biofilms are associated with a host, chemical gradients and their origins become highly relevant. Conventional biofilm laboratory models such as multiwall biofilm models and agar plate models poorly mimic these gradients. In contrast, ex vivo models possess the partial capacity to mimic the conditions of tissue-associated biofilm and a biofilm associated with a mineralized surface enriched in inorganic components, such as the human dentin. This review will highlight the progress achieved using these settings for two models of persistent infections: the infection of the lung tissue by *Pseudomonas aeruginosa* and the infection of the root canal by *Enterococcus faecalis*. For both models, we conclude that the limitations of the conventional in vitro systems necessitate a complimentary experimentation with clinically relevant ex vivo models during therapeutics development.

## 1. The Formation of Persistent Infection in a Host

Persistent infections can be defined as communities of bacteria that do not belong to the natural flora of a given host and colonize a tissue or a surface. As these communities adhere to the natural/foreign surface, they are frequently defined as a biofilm [1,2]. This definition is quite accurate, as the formation of extracellular polymers that generate a complex matrix by the biofilm cells plays a cardinal role in host colonization [2,3,4]. For example, the enteropathogens *Escherichia coli* and *Salmonella* secrete autoaggregative curli fibers that mediate cellular aggregation, host colonization, and pattern formation [5,6,7]. Similarly, the overproduction of the exopolysaccharide alginate by mucoid *Pseudomonas aeruginosa* strains is associated with higher morbidity and mortality in patients with cystic fibrosis (CF), a genetic disorder that leads to defective lung function, sputum accumulation, and increased susceptibility to *P. aeruginosa* infections [8]. The alginate-containing biofilm produced by the mucoid strains increases the tolerance of the biofilm cells to antibiotics and phagocytosis of bacteria. In agreement, treatment with the enzyme alginate lyase improves antibiotic responses and the performance of the lungs as judged by sputum viscosity [9,10].

Cells of an aggregate/film that is associated with a tissue are placed in a bidirectional open system with different nutrients provided by the tissue and its surrounding fluids, as well as an oxygen and trace elements supply [11]. Tissues enriched with inorganic components (e.g., dentin and apatite) can be gradually degraded by the acidic products of microbial fermentation [12] that are prone to produce local calcium and phosphate concentrations.

Therefore, gradients of ions and molecules with spatial distribution altering along the tissue from their origins (high) to distal areas (low) are highly relevant to biofilm physiology. Conventional biofilm laboratory models, such as a flow cell, where bacteria are grown on a plastic/glass surface under flow, multiwall biofilm models, and agar plate models, poorly mimic the local conditions [11,13,14,15]. Although each in vitro model has its limitations, collectively they promoted significantly our understanding of biofilm biology [11,13,14,15]. To complement the finite resolution of an in vitro model, ex vivo models can partially mimic the conditions of both tissue-associated biofilm [16] and abiotic-surface/mineral-associated biofilms [17].

We note that the colonization of a medical device generates different topology of nutrients flow: one side of the film provides little or no nutrients and gases, and the other side (tissue), is rich in both. Devise-associated biofilms areeasier to mimic in laboratory settings. In a host, the tissue is a dynamic provider of nutrients that are essential to promote the growth of the bacteria., The tissue is also a source for immune components (antibodies and immune cells) targeting the infection site [18]. Interestingly, geochemical gradients are primarily considered while characterizing environmental biofilms, such as river communities, soil communities, and even plant (host)-associated communities [19,20,21]. In contrast, chemical gradients are frequently understudied in the context of a mammalian host. The poor capacity to map chemical gradients in vivo may account for this lack of symmetry [22,23].

In this review, we will expand on two specific ex vivo model systems of persistent infections, keeping most of the spatio-temporal features of host-associated communities: the first model mimics *P. aeruginosa* infections in the lungs of compromised patients or on top of a medical device. This is the most characterized model for persistent biofilm infection [22]. The second model studies the Gram-positive pathogen *Enterococcus faecalis*, which generates persistent biofilm infections formed over the mineralized dentine surface of the tooth, within the root canal, and on top of medical devices [24]. Within this habitat, *E. faecalis* is by far the most characterized biofilm former. These Gram-negative and Gram-positive pathogens differ in their extracellular matrix, biofilm genes, and their regulators as discussed below, but they similarly reflect the contribution of ex vivo systems to investigate infections and their treatment protocols. According to Webster’s Dictionary, ecology is “the totality or pattern of relations between organisms and their environment”. One fundamental component of the immediate environment of a microorganism is surface topography: e.g., the different configurations of the surface in contact with the microorganism. Here, we will try to apply this notion while discussing the laboratory models of host-associated infections.

## 2. In Vitro Laboratory Models to Study Biofilms

In general, biofilm laboratory models for biofilm infections can be classified into main categories: in vitro, ex vivo, microcosms, organ-on-a-chip modes, and in vivo models. In all of the above, various microorganisms can be cultivated.

Basic in vitro models can mimic anaerobic and aerobic environments. They are instrumental to evaluate the adherence of defined microorganisms to a surface, which can be either abiotic (typically, polystyrene, glass or polypropylene, or even titanium to mimic medical implants) or biotic (commonly resin, or alginate beads). These defined systems can also be used to investigate the efficiency of antibiotics and antimicrobial substances, and to mimic the treatment strategies to prevent the formation of biofilms or disturb the pre-established biofilms [25]. In vitro models can be either static or dynamic.

**Static in vitro biofilm models:** Allow quantification of biofilm species interacting within the settings to assess the overall biomass of the formed biofilms, the chemical composition of the secreted exopolymers (EPS), and the overall structure of the extracellular matrix (ECM).

**Dynamic in vitro biofilm models:** Unlike the static model, where nutrients are depleted and waste products are accumulated, in a dynamic model, fresh media is constantly provided and dead cells and waste products are continuously removed. Dynamic models include the flow cell systems and chemostats.

## 3. The Limitations of In Vitro Models to Mimic Infection

In vitro models are limited in their capacity to mimic the complex in vivo environment. For example, there is a distinct difference in the transcriptomic profiles between *P. aeruginosa* isolates grown in artificial synthetic sputum [26] in vitro and a direct analysis of the microbial transcriptome from CF sputum samples. For example, the overproduction of genes associated with microbial communication and the production of the polysaccharide alginate, associated with a mucoid phenotype of this bacterium was observed in vitro [27,28]. This indicates that although the artificial sputum closely reflects the CF lung environment, it fails to mimic the metabolic microenvironment of the biofilm. In terms of the biofilm topography, cells and matrix seen in microtiter plates are homogenous and compact and, therefore, are strikingly different from the “mushroom” structures and open channels observed in flow cells [29]. However, lung biopsies of people with CF do not resemble either of the models as they reveal biofilms suspended in bronchial mucus with a “sponge”-like appearance. The gaps between the cells are filled with microbial alginate, human or microbial mucus, and lung fluid [30]. Hence, it is likely that in vitro biofilms do not accurately mimic CF biofilms or the structure-dependent aspects of infection discussed previously [11]. The limitations of in vitro models were discussed previously in additional systems, including device-related infections [31,32].

## 4. Realistic Laboratory Models of Infection

**Microcosms:** Microcosms are elegant models, aimed to accurately mimic both environmental and clinical conditions. These models include a large repertoire of microbial community members and apply realistic substrates. For example, a microcosm model for oral infection will rely on saliva and hydroxyapatite to model biofilms. Layering abiotic surfaces, as well as human cells, can highly resemble the relevant clinical situations [33]. Ideally, both static and dynamic systems can be converted into microcosms. However, while the complexity of the initial community reflects the natural conditions, frequently the setting of the microcosm model itself is highly artificial—for example, conventional growth media lacks the spatial features of the origin niche [34]. It is interesting to note that for persistent infections of CF, the combination of an artificial sputum medium and a microfluidic device based on a narrow capillary allows an oxygen gradient and growth conditions similar to those that exist in mucus bronchioles [35].

**Ex vivo tissue culture:** Cultured mammalian cells poorly mimic the surface-bacteria interactions of an infection as the three-dimensional organization of solid tissue consists of a variety of cells and unique host extracellular matrix components. In contrast, ex vivo organ culture systems from mouse, human, and alternate host tissues keep the original topography of the tissue cultures, and their viability can be maintained for several days. The ex vivo system critically depends on proper slicing and culturing in optimized media of the sliced tissue [36,37,38,39]. In general, the segments of tissues that can maintain their viability over days are relatively large (mm scale) and need to be maintained appropriately (e.g., six-well plates and similar). However, these systems can be converted to microphysiological systems (MPS) (96-well or 384-well plates) once a technique to maintain the viability of small tissue fragments becomes available. The term “MPS” is applied generally to all “micro-sized” physiological living systems, with various layers of overlapping terms describing the systems that can be used for in vitro testing [40]. These terms can either apply or not to the ex vivo lung model, depending on the size of the settings.

**Ex vivo models for dentin-associated oral infections**: Dentin sections are prepared and sterilized. In short, the crowns of the selected teeth can be removed to obtain multiple root specimens. To prepare the dentin slabs, the roots are cut perpendicular to the long axis of the root under water cooling and sterilized overnight using ethylene oxide gas [17]. Two ex vivo systems are frequently used to analyze *E. faecalis* infections in vitro—the dentin slabs model that mimics the interaction of bacteria with dentin [17] and a root-canal infection model that mimics the root canal colonization [41].

**Organs on Chips:** Organs on chips are formed by human cells placed on top of microfluidic devise, and can mimic the complex structures and functions of human organs and thereby mimic human organ-specific responses [42,43]. The first lung-on-chip model was introduced in 2010 [44]. Since then, chip-based disease models became important research tools in numerous applications and specifically in studying viral infections [42]. Their limitations include a relatively high complexity compared with 3D ex vivo models and a failure to mimic infections associated with abiotic surfaces, e.g., bones or teeth, as they cannot form de novo in the device.

## 5. Case Studies for the Ex Vivo Studies of Biofilm Infections

### 5.1. P. aeruginosa

The biofilm biology of this pathogen is studied in depth and its genetics is fairly well-understood: the extracellular matrix of *P. aeruginosa* biofilms in vitro depends heavily on exopolysaccharides and extracellular DNA (Edna). The three exopolysaccharides, i.e., Psl, Pel, and alginate, are vital for surface attachment, formation, and the assembly of the 3D structure of the biofilm: Psl is a neutral pentasaccharide comprising d-glucose, d-mannose, and l-rhamnose. When the biofilm is mature, the Psl is located at the borders of the mushroom-like structure. Psl is essential for the stability of mature biofilms [45]. Increased Psl expression promotes the aggregation of cells in liquid, a phenotype associated with the sputum of CF patients [46]. Pel is a cationic polysaccharide polymer, the subunits of which include deacetylated N-acetyl-d-glucosamine and N-acetyl-d-galactosamine. Pel is important for initial surface attachment, as well as for maintenance of biofilm integrity. Pel is also essential for the formation of floating biofilms in the water–air interface [47]. Pel promotes the tolerance to aminoglycoside and to the antibiotic colistin [48]. In addition to Pel and Psl, the polysaccharide alginate is predominately produced in mucoid Pseudomonas isolates found in CF patients because of a mutation in mucA22 allele [46]. The production of alginate plays an important role in biofilm maturation and during interaction with the immune system as alginate protects biofilm residents from both phagocytosis and opsonization. In addition, alginate presence is correlated with a decreased diffusion of antibiotics through the biofilm. Cell lysis releases eDNA supporting cellular organization and twitching motility [49].

The chronic infection of *P. aeruginosa* is characterized by the embedding of biofilm aggregates in a host mucus found in the airways of people with CF. Once established, there is a low correlation between antibiotic susceptibility testing and the outcome of treating the patient with antibiotics, indicating a failure of the in vitro system to predict the result in vivo. To overcome the gaps between the clinic and the laboratory and to develop effective treatments versus biofilm infections, the development of novel models to study clinically realistic, CF-associated chronic biofilm infections in the laboratory is indispensable. To complement the in vitro studies, and validate biofilm biology in a clinically relevant scenario, an ex vivo pig lung (EVPL) model [30] for *P. aeruginosa* CF lung infection can be used. To mimic infection, an ex vivo model combines one formulation of artificial sputum medium with sections of porcine bronchiole as pig lungs demonstrate a close similarity to human lungs. *P. aeruginosa* metabolism and biofilm formation were highly similar to the status of CF lung. Biofilm structures were also highly conserved [30]. In addition, the antibiotic tolerance of *P. aeruginosa* CF isolates grown as biofilms ex vivo was enhanced similarly to clinically relevant scenarios. To support clinical relevance, the transcriptome of biofilm cells grown ex vivo unraveled the existence of topography and surface sensing by the cells; two distinct growth environments were observed from the transcriptome: tissue-associated biofilm and detached cells in its proximity, each cuing a transcriptome distinct from that seen in vitro. The expression of quorum-sensing-associated genes in tissue-associated biofilm was similar to CF sputum versus in vitro [27]. Thereof, ex vivo is superior to alternate models in mimicking chronic infection conditions.

An ex vivo system also allows the consideration of therapeutics delivered in a clinical scenario, e.g: within a tissue. The gas nitric oxide (NO) is a known signal for biofilm dispersal in *P. aeruginosa* and is frequently suggested as a potential therapy for its infections [50]. However, the lung tissue substitutes a significant barrier toward NO in accessing the biofilm cells. Therefore, the ex vivo system was used to demonstrate that the bactericidal action in the tissue of the chitosan with NO-releasing capacities was greater compared to NO alone. Hence, in realistic conditions and in tissue, delivery of NO by chitosan is superior to direct gas with bactericidal and mucolytic action [51].

A similar approach aiming to interfere with the microenvironment of the infection is an intervention in microbial mineralization and carbonate production. Recent studies highlighted a role for biominerals in biofilm development. The pathogens *Proteus mirabilis*, *Proteus vulgaris*, and *Providencia rettgeri* generated calcium and magnesium minerals on catheters [52]. Confocal microscopy detected minerals in in vitro biofilms, dominated by calcium carbonate [53], and carbonic anhydrase [54] was shown to mediate calcium deposition in *P. aeruginosa*. In addition, in *P. aeruginosa*, calcium was found to mediate cross linking of the exopolysaccharides of the mucoid biofilms independently of Psl [55]. Using an ex vivo system from the murine host, lung cell death during infection by *P. aeruginosa* was inhibited with carbonic anhydrase and calcium uptake inhibitors, and, with mutants lacking carbonic anhydrases (CAs), enzymes that mediate the conversion of interconversion between carbon dioxide and water to carbonic acid (i.e., bicarbonate and hydrogen ions). This work suggested several new categories to combat lung infections. Furthermore, as no single treatment was significantly more effective than others in all tested models, all biochemical processes contributing to biomineralization are likely of crucial importance to host infection. In this work, the effects of carbonic anhydrase, urease (contributing to carbonate production), and calcium uptake on biofilm formation in vitro and tissue colonization ex vivo were robust. Still, their relative contributions under each setting differed [16].

### 5.2. E. faecalis

The formation of pili to facilitate cell adhesion is also essential for biofilm formation. In addition, the absence of genes encoding biofilm-associated pili genetic locus (EbpABC) and the downstream sortase (Srt) (the transpeptidases that attach the secreted proteins to the peptidoglycan cell wall) hampers biofilm formation. Also contributing to biofilm formation are the quorum-sensing system, Fsr (fecal streptococci regulator), which is comprehensively studied, and peptide pheromones secreted by recipient cells to induce the conjugative apparatus of donor cell as they promote intercellular communication of *E. faecalis* cells within the biofilm [56].

For dental treatments, ex vivo biofilm models are increasingly used to predict the outcome of treatment and irrigators. However, their structure, gene expression profiles, and essential infection genes remain to be determined. As the surface in root-canal models is frequently human teeth, ex vivo models are the closest to the ecology and surface topography of the infection site. Ex vivo mechanistic studies (as done for *P. aeruginosa)* are vital to develop and adjust updated therapeutic strategies for persistent dental infections.

So far, ex vivo studies were primarily used to determine the effectivity of existing treatments. For example, the limitations of sodium hypochlorite as an irrigator of *E. faecalis* pre-established biofilms were described in the dentin-association model [17]. This study offered D-amino acids, cell wall modulators [57,58], as an alternate appealing therapy to deal with persistent infections. Similarly, *E. faecalis* was recovered from chlorhexidine irrigation and immediate root filling in a single visit and a combinatory treatment of chlorhexidine irrigation, filling after 14 days, and a use of a calcium hydroxide dressing in multiple visits. The effectivity of the treatments was assessed ex vivo, and the results were distinct from in vitro models as both treatments were sufficient as antimicrobials in vitro. Importantly, all root canal treatments ex vivo failed to eliminate *E. faecalis* completely from dentinal tubules [59]. These results highlight a need to investigate additional “out of the box” therapies aiming to eradicate *E. faecalis* in full from the infected canals, one being probiotic strains and their products [60].

Ex vivo systems are also frequently used to evaluate the effectivity of root canal sealers. Bacteria can colonize the interface between the root canal sealer and the canal wall (as evaluated in [61]), or to penetrate deep into the dental tubules [41]. The irrigation protocol may affect the ability of root canal sealers to prevent bacterial colonization in the filling–dentin interface. However, irrigations cannot penetrate into the tubules where persistent bacteria are highly clinically relevant.

In contrast to the above, the ex vivo system also allows us to determine when the clinical protocols are in a higher dosage than recommended. Using an ex vivo system, it was shown that a TAP treatment (ciprofloxacin, metronidazole, and doxycycline) used to eliminate *E. faecalis* from the root canal is used at 50,000 times its MIC (minimal inhibitory concentration). Considering an increased risk of coronal discoloration of teeth following the use of this formulation and subsequent aesthetic defects for the patients [62], the results of this study call for a re-evaluation of the current working concentrations of TAP.

Overall, the relatively extensive study of *E. faecalis* ex vivo highlights the potential of an ex vivo study to adjust and improve the working concentrations and treatments in clinically relevant protocols.

## 6. Disadvantages of the Ex Vivo Models

Throughout the review, we emphasized the numerous benefits of the ex vivo models, representing together with microcosms and organs-on-a-chip, a more holistic approach to studying microbial infections. However, several obstacles are associated with choosing these models. Most importantly, the experimenters need to consider the source of the harvested tissue. If it is taken from an animal model (e.g., murine or pig for the ex vivo lung), the model is prone to suffer from the same shortcoming of animal experiments. An animal trial presents an ethical problem, and the relevance of animal tissues for drug development needs to be determined on a case–case base because of the differences between the model animals and human hosts.

Unlike conventional animal models, the harvest of multiple tissue replicates from a single-animal decreases significantly the number of animals sacrificed per experiment. In these models, the resolution of the host and the pathogen is comparable to an in vitro model. In addition, the methodologies for harvesting ex vivo tissues are reasonable and well-established [37,39,63,64]. Harvesting tissues from a human host reduces the ethical complexity (as tissues are usually harvested during biopsies) but markedly leads to variation between extracted tissues. Unlike laboratory models and clinical trials, the random selection of human tissues could affect the significance of the results. In addition, human tissues from healthy individuals are not readily available for ex vivo lung infection models. In contrast, extracted human teeth are available from the clinic and can be easily handled making the ex vivo study of oral infection relatively uncomplicated and straightforward. Although access to human tissues is limited and animal tissues represent ethical and physiological challenges, the ex vivo models narrow the gap between pre-clinical and clinical studies and increase the chances of a successful drug discovery.

## 7. Summary

**Figure 1:**
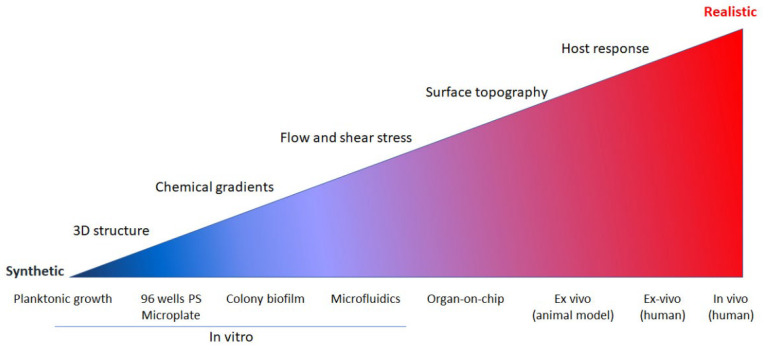
Represented are the relative relatedness of laboratory model systems to in vivo infection, from the most distinct to highly related (red-realistic, blue-synthetic). We note that all systems are informative and with considerable scientific merit. For example, the multiplate essay is highly compatible with high throughput systems. The gradient represents how the model mimics the abovementioned factors for microbial infections.

Biofilm models are designed to properly estimate the viability of the community members (dead/living cells), the thickness (monolayered or multilayered) of the biofilm, and its overall structure with an emphasis on density, homogeneity, regularity, density, and surface topography. Nevertheless, artificial settings often used in vitro fail to mimic the hosts’ effect on biofilm fundamental properties such as gene expression, drug resistance, and extracellular matrix composition. The inconsistencies of laboratory settings with infection biology are detailed in Figure 1.

Examples are the formation of homogenous biofilms of *P. aeruginosa* on a multiwall plate and mushroom-like bodies by cells grown in a flow chamber. None of these structures are representative of biofilms formed in the lungs of CF patients, which are generally sponge-like aggregates. The effect of an extracellular matrix from the host such as mammalian mucin used by biofilm cells and promoting antibiotic tolerance in vivo is absent from in vitro models [65,66].

Similarly, the biofilm cells of the pathogen *E. faecalis* in the root canal are influenced by the unique topography of the exposed canal and gradients of minerals from demineralization of the calcium phosphate surface that surrounds it and different nutrients from saliva. While mimicking infection under laboratory conditions is not trivial, ex vivo systems are considered a reasonable solution to narrow the gap between the clinic and the lab.

Within this review, we demonstrated how these systems allow the predicting of the outcome of persistent biofilm infections and adjusting potential therapies for these infections.
